# A multicentre validation study of a smartphone application to screen hand arthritis

**DOI:** 10.1186/s12891-022-05376-9

**Published:** 2022-05-09

**Authors:** Mark Reed, Broderick Rampono, Wallace Turner, Andreea Harsanyi, Andrew Lim, Shereen Paramalingam, David Massasso, Vivek Thakkar, Maninder Mundae, Elliot Rampono

**Affiliations:** 1 Perth, Australia; 2 Hollywood Medical Centre, Suite 41, 85 Monash Avenue, Nedlands, Western Australia Australia; 3 Sydney, Australia; 4 Melbourne, Australia

**Keywords:** Early arthritis, Screening, Diagnosis, Machine learning, Artificial intelligence, Telemedicine

## Abstract

**Background:**

Arthritis is a common condition, and the prompt and accurate assessment of hand arthritis in primary care is an area of unmet clinical need. We have previously developed and tested a screening tool combining machine-learning algorithms, to help primary care physicians assess patients presenting with arthritis affecting the hands. The aim of this study was to assess the validity of the screening tool among a number of different Rheumatologists.

**Methods:**

Two hundred and forty-eight consecutive new patients presenting to 7 private Rheumatology practices across Australia were enrolled. Using a smartphone application, each patient had photographs taken of their hands, completed a brief 9-part questionnaire, and had a single examination result (wrist irritability) recorded. The Rheumatologist diagnosis was entered following a 45-minute consultation. Multiple machine learning models were applied to both the photographic and survey/examination results, to generate a screening outcome for the primary diagnoses of osteoarthritis, rheumatoid and psoriatic arthritis.

**Results:**

The combined algorithms in the application performed well in identifying and discriminating between different forms of hand arthritis. The algorithms were able to predict rheumatoid arthritis with accuracy, precision, recall and specificity of 85.1, 80.0, 88.1 and 82.7% respectively. The corresponding results for psoriatic arthritis were 95.2, 76.9, 90.9 and 95.8%, and for osteoarthritis were 77.4, 78.3, 80.6 and 73.7%. The results were maintained when each contributor was excluded from the analysis. The median time to capture all data across the group was 2 minutes and 59 seconds.

**Conclusions:**

This multicentre study confirms the results of the pilot study, and indicates that the performance of the screening tool is maintained across a group of different Rheumatologists. The smartphone application can provide a screening result from a combination of machine-learning algorithms applied to hand images and patient symptom responses. This could be used to assist primary care physicians in the assessment of patients presenting with hand arthritis, and has the potential to improve the clinical assessment and management of such patients.

**Supplementary Information:**

The online version contains supplementary material available at 10.1186/s12891-022-05376-9.

## Background

Arthritis is a common condition and is the most frequent cause of disability in US adults. In 2013, the impact of arthritis on the US health budget was in excess of $300 billion in direct and indirect costs [1, 2]. CDC data from 2021 revealed that 58.5 million (23.7%) US adults have arthritis, 25.7 million (43.9%) of whom have arthritis-attributable activity limitations [3]. Other sources have indicated that this may reflect a significant under-estimation of the true number of arthritis cases, for which the prevalence is projected to increase by 49% from 2010 to 2040 [4, 5]. A national health survey from 2017 to 18 estimated that 3.6 million (15%) of Australians suffered from arthritis, excluding back pain; rheumatoid arthritis was estimated to affect 1.9% of the population [6].

The most common form of arthritis is osteoarthritis (OA), followed by inflammatory arthritis (IA). Among IA, rheumatoid arthritis (RA) and psoriatic arthritis (PsA) are the two most common subtypes. The presentation of both OA and IA is commonly related to signs and symptoms in the hands. Significant progress has been made with IA treatment over the past two decades, including biological and targeted synthetic disease modifying medication. In contrast, screening methods have not evolved, leading to significant delays in accurate identification and treatment, compounding the burden of disability [7–9]. There is a need for reliable, accessible and cost-effective methods to help identify IA in primary care settings.

Since the COVID-19 pandemic began, there has been enhanced uptake of digital health methods, with limited face-to-face medical appointments and increased reliance on online and virtual platforms [10–12]. Machine learning (ML) has been applied to various fields of medicine, and its application has been accentuated by the increasing use of Telemedicine [13]. ML is a statistical technique incorporating sample datasets on which a model is trained, to develop predictions without explicit programming [14]. ML can employ a range of different algorithms including neural networks, deep learning, decision trees, support-vector machines, and Bayesian networks.

ML techniques have been applied to several aspects of rheumatology, including electronic health records, imaging, disease classification, disease outcome and treatment response prediction [15, 16]. Previous ML applications to diagnose rheumatological conditions have used biological samples, such as serum biomarkers and genomic data from synovial tissue [17, 18]. ML has also been applied to ultrasound techniques to enable a computer-aided diagnosis, for rheumatoid arthritis, osteoarthritis and systemic lupus erythematosus [19]. Such approaches continue to be limited by practical considerations, including accessibility, cost and time, in addition to variable accuracy.

We have developed and tested an arthritis screening tool to assist in the assessment of patients presenting with hand arthritis [20]. The tool combined ML algorithms applied to photographic images, survey results and a single examination technique. The results of the pilot study showed significant promise of the screening tool to distinguish between OA, RA and PsA, or a combination of these conditions.

The aim of the present study, was to examine the performance of the screening tool among a number of different Rheumatologists across Australia. Our hypothesis was that the outcomes of the multicentre analysis would be comparable to the pilot study, eliminating the potential for single-user bias, and confirming the validity of results across different Rheumatology practices.

## Methods

Rheumatologists from 10 different practices around Australia were invited to take part in the study. Participating Rheumatologists were required to have a minimum of 3 years’ experience as consultant, and were asked to recruit a minimum of 10 and maximum of 60 patients for this study. Recruiting of new patients was limited in some regions due to COVID-19 outbreaks, restricting access to face-to-face appointments. At the conclusion of a new patient appointment, each patient presenting with arthritis affecting the hands was invited to participate in the study. Any new adult patient with hand arthritis was eligible to enrol.

Following on from the pilot study, an integrated smartphone application (App) was developed incorporating photographic capture of the patient’s hands, followed by survey and examination results. If agreeable, the patient was required to tick a consent statement within the App, with age and sex being recorded. A photograph of the dorsal aspect of both left and right hands were taken, and a 9-part survey was completed. The Rheumatologist provided a single examination result- wrist irritability – referring to the presence or absence of pain on passive wrist flexion. The doctor’s clinical diagnosis was then entered, based on the patient’s history, examination, and investigation findings from a 45-minute consultation. Each Rheumatologist was provided with written instructions and example images outlining the technique for taking photographs of the hands, preferably on a white background, with the wrist exposed.

The screening result of the App was not available to the clinician at the time of data capture. Components of the survey, and the process of the algorithm development and initial testing, have been reported previously [20]. A total of 248 consecutive new patients presenting to 7 Rheumatologists in private practices across Australia were included in the study, between March 2020 and March 2022.

An ensemble of five different ML models were trained as part of the arthritis prediction pipeline. Through transfer learning techniques, the pre-trained VGG-16 [21] convolutional neural network (CNN) was used as the base layer for training two image pre-processing models (model A and model B) on 1577 diagnosis-labelled hand images. These models were responsible for rotating and horizontally flipping hand images to a standard orientation. A similar technique, in conjunction with image augmentation methods, used 1013 hand images to train the classifier model responsible for predicting the diagnosis of OA from an oriented hand image (model C).

**Fig. 1:**
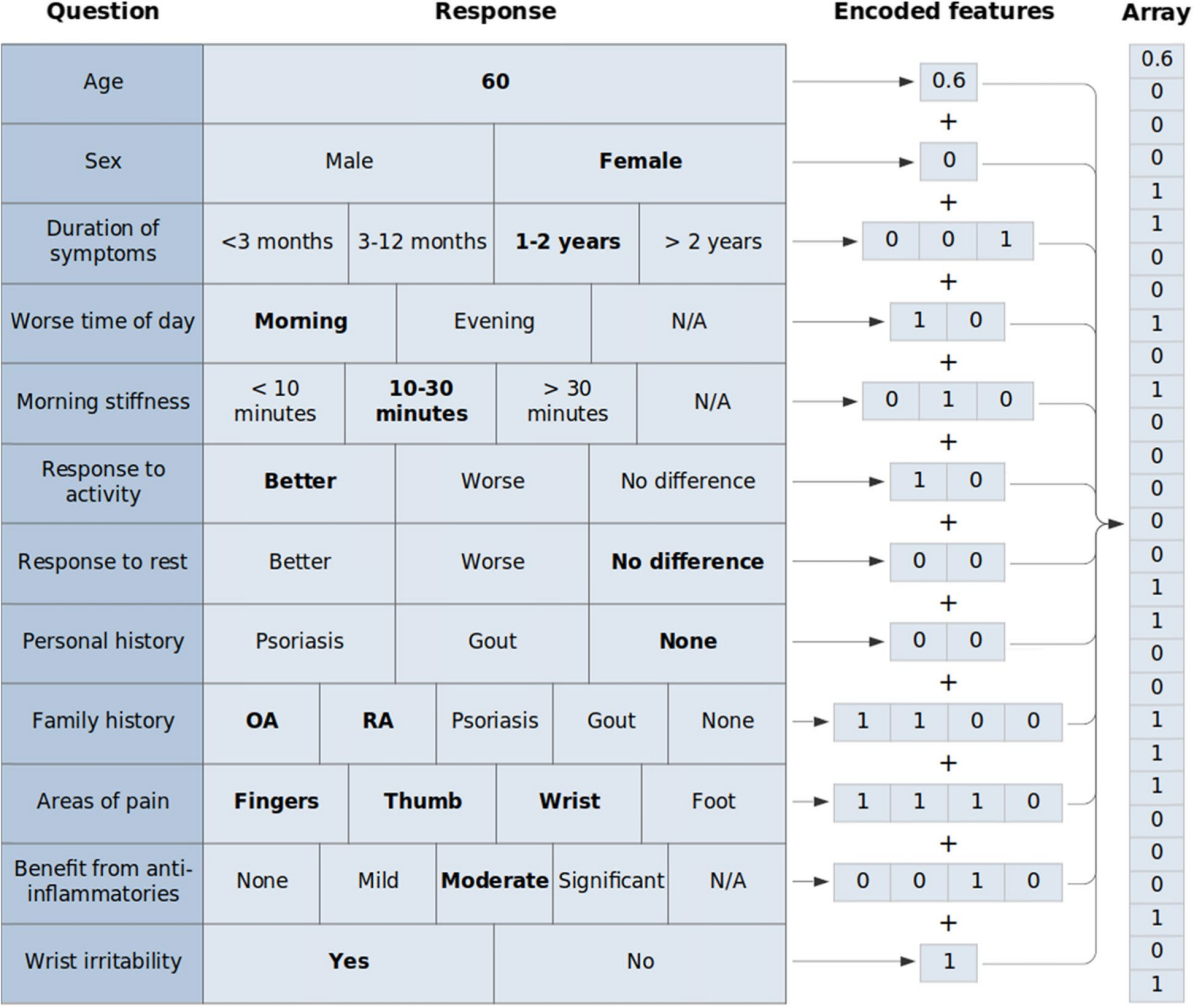
Example survey feature extraction using one-hot encoding of categorical features and scaling of numerical features. Positive survey responses are shown in bold

**Fig. 2:**
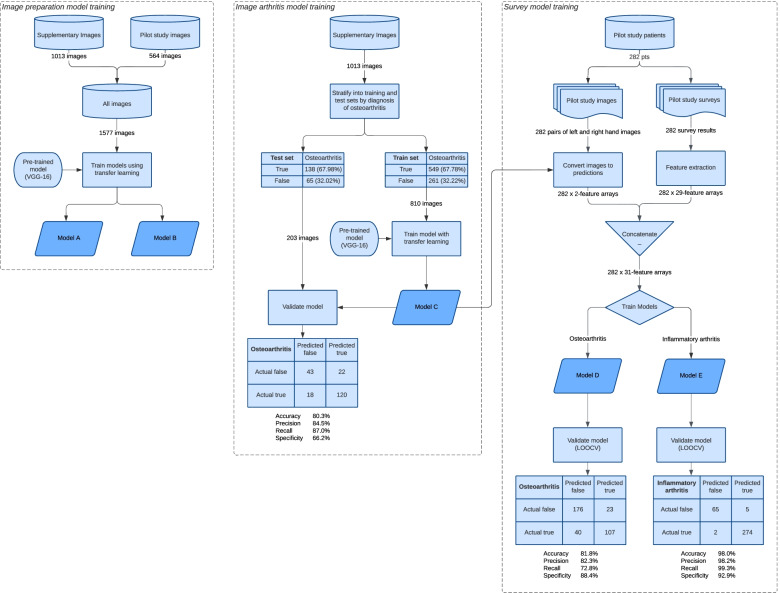
Machine learning model training pipeline

Combined survey and image data from 282 patients were processed into a feature array by one-hot encoding categorical responses and scaling numerical responses (Fig. 1). Hand images were then converted to a left-hand and right-hand prediction using model C and appended to this array. A gradient boosting classifier was trained on these 282 processed arrays to produce OA (model D) and IA (model E) prediction models. The model training pipeline is summarised in Fig. 2.

**Fig. 3:**
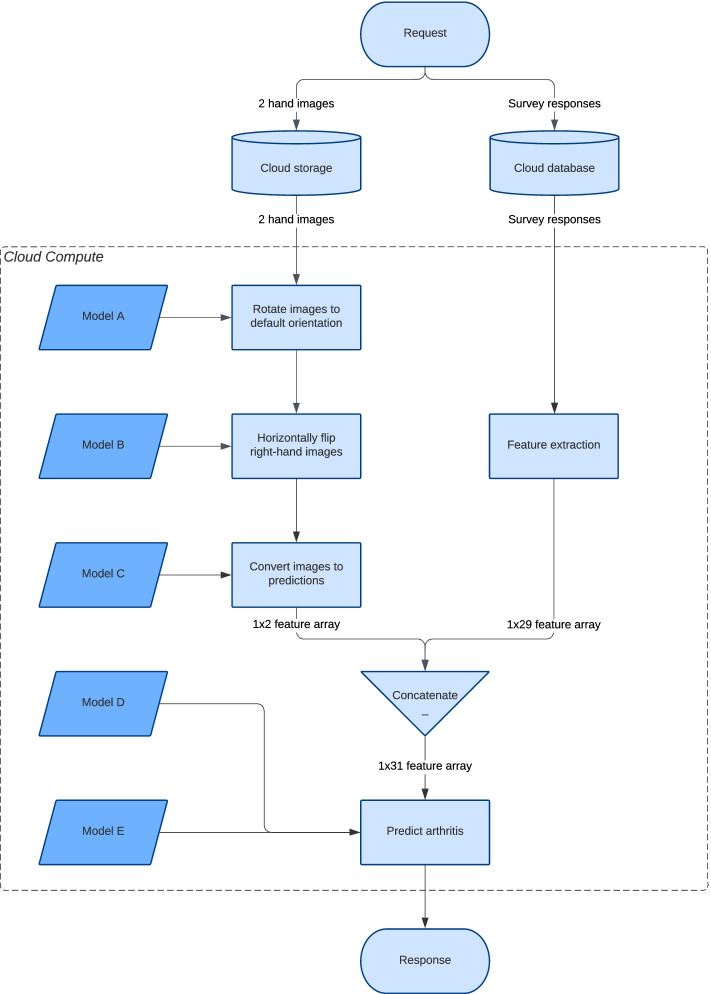
Online model prediction endpoint

The models were hosted online and integrated into a mobile App using Google Cloud Platform. The process of converting a user’s image and survey data to an arthritis prediction is presented in Fig. 3. Patients with an IA prediction were converted to an RA or PsA prediction based on the absence or presence of psoriasis in the patient’s personal or family history. A personal history of current or prior psoriasis was provided by the patient within the survey, as was a family history in first degree relatives.

Four metrics of predictive power were used to evaluate each model: accuracy, precision, recall, and specificity. Accuracy reflects the total proportion of all correct model predictions, precision (positive predictive value) refers to the proportion of diagnosis-positive predictions that were truly diagnosis-positive, and recall (sensitivity) describes the proportion of all truly positive diagnoses that the model was able to correctly identify. Conversely, specificity indicates the proportion of all truly negative diagnoses that the model was able to correctly identify.

## Results

The patient cohort’s demographic features and clinical diagnoses are presented in Tables 1 and 2. 66.9% of the patients were women, with a mean age of 60.1 years. The most frequent diagnosis was OA (95), followed by RA (79), coexistent OA/RA (30), PsA (24) and coexistent OA/PsA (9).

**Table 1:** Diagnostic summary of all patients

Rheumatologist Diagnosis Number (percentage)					
OA	RA	PsA	OA & RA	OA & PsA	Other
95 (38.3%)	79 (31.9%)	24 (9.7%)	30 (12.1%)	9 (3.6%)	11 (4.4%)

**Table 2:** Diagnostic summary stratified by Rheumatologist

Rheumatologist	Number of cases	Mean age (years)	Rheumatologist Diagnosis					
			OA	RA	PsA	OA & RA	OA & PsA	Other
Dr A	60	56.7	25.0%	36.7%	18.3%	18.3%	1.7%	0.0%
Dr B	60	62.8	58.3%	28.3%	1.7%	3.3%	5.0%	3.3%
Dr C	60	58.3	23.3%	30.0%	15.0%	21.7%	3.3%	6.7%
Dr D	22	62.5	50.0%	13.6%	4.5%	13.6%	9.1%	9.1%
Dr E	20	62.5	75.0%	25.0%	0.0%	0.0%	0.0%	0.0%
Dr F	14	58.5	14.3%	42.9%	7.1%	7.1%	7.1%	21.4%
Dr G	12	66.8	25.0%	66.7%	8.3%	0.0%	0.0%	0.0%

The application’s performance with regard to predict each diagnosis are presented in Table 3.

**Table 3:** Case counts and evaluation metrics of arthritis predictions for each single diagnosis

Diagnosis	Number of cases	Accuracy	Precision	Recall	Specificity
OA	134	77.4%	78.3%	80.6%	73.7%
RA	109	85.1%	80.0%	88.1%	82.7%
PsA	33	95.2%	76.9%	90.9%	95.8%

Secondary analysis found that evaluation metrics were similar in the absence of each contributing Rheumatologist, confirming that the results were not substantially influenced by a single user’s performance (Table 4).

**Table 4:** Case counts and evaluation metrics of arthritis predictions with each individual doctor excluded from analysis

Doctor Excluded	Diagnosis	Number of cases	Accuracy	Precision	Recall	Specificity
Dr A	OA	107	76.1%	77.7%	81.3%	69.1%
	RA	76	82.4%	74.7%	85.5%	80.4%
	PsA	21	94.7%	72.0%	85.7%	95.8%
Dr B	OA	94	76.6%	76.6%	76.6%	76.6%
	RA	90	85.6%	82.5%	88.9%	82.7%
	PsA	29	95.7%	81.8%	93.1%	96.2%
Dr C	OA	105	78.7%	78.3%	85.7%	69.9%
	RA	78	83.5%	77.0%	85.9%	81.8%
	PsA	22	95.2%	74.1%	90.9%	95.8%
Dr D	OA	118	77.0%	76.6%	80.5%	73.1%
	RA	103	87.6%	85.0%	88.3%	87.0%
	PsA	30	95.6%	77.8%	93.3%	95.9%
Dr E	OA	119	76.8%	77.0%	79.0%	74.3%
	RA	104	85.1%	80.7%	88.5%	82.3%
	PsA	33	95.2%	78.9%	90.9%	95.9%
Dr F	OA	130	78.6%	80.8%	80.8%	76.0%
	RA	102	85.5%	79.8%	89.2%	82.6%
	PsA	31	94.9%	75.7%	90.3%	95.6%
Dr G	OA	131	78.0%	80.2%	80.2%	75.2%
	RA	101	85.2%	78.9%	89.1%	82.2%
	PsA	32	94.9%	76.3%	90.6%	95.6%

The median time for data capture, from the time of the patient’s consent until the doctor’s diagnosis was entered, was 2 minutes and 59 seconds (2:59). This value remained similar in the absence of any single contributor from the analysis, with the median time between 2:40 and 3:35.

## Discussion

The results of this study confirm that this App can accurately discriminate between different forms of hand arthritis, with consistent results across a number of different Rheumatologists. The App provides a simple, reliable, and rapid screening test for patients presenting with arthritis affecting the hands. It can provide a point-of-care result to primary care physicians (PCPs), reducing the need for separate investigations, including blood tests and imaging. This approach could improve accuracy compared to traditional screening methods, and significantly reduce patient and healthcare costs.

The median time taken for data capture, at less than 3 min, could be readily incorporated into a brief primary care consultation. The ease of use and accuracy compares favourably to other screening techniques with more complicated scoring systems [22–24]. Several such tools include blood tests and imaging results, with associated additional time and costs [23–25].

Previous studies have confirmed significant delays in specialist review and treatment for patients with IA [7–9]. The majority of irreversible damage in RA occurs in the early stages of the disease, and patients presenting with longer disease duration are less likely to achieve long-term drug-free remission [26, 27]. Delayed diagnosis and treatment of PsA has also been correlated with poorer physical function and radiographic outcomes [28]. Timely identification and referral of patients with IA from primary care providers form an integral part of this delay [29].

A case-review analysis reported a median delay of 161 days, and a median of 5 visits, for specialist referral of RA patients after primary care presentation [30]. During the early stages of presentation, 82% of RA patients were not considered by their PCP to have an inflammatory pathology. Interviews that incorporated patient perspectives, identified frustration in delays for specialist referral, and concerns that their symptoms were misinterpreted or neglected [31, 32]. In contrast to the critical window for IA treatment, patients with isolated OA do not require a formal specialist review, as there are no disease-modifying treatments for this condition [33]. Data from early-arthritis clinics has shown that similar proportions of patients with OA and RA were referred for assessment [34, 35]. Comparable results were seen in this study, with 38.3% of patients referred for Rheumatologist review having OA alone. This could be reduced by a screening programme that allows PCPs to confidently identify and manage patients with isolated OA, and to better recognise and refer patients presenting with IA.

Traditional screening for arthritis in primary care relies on blood tests and imaging, which have been shown to have limited sensitivity, particularly in early disease [36–40]. The absence of a reliable blood test for psoriatic arthritis further reduces the capacity for such patients to be identified. Patients with established OA who develop coexistent IA represent a more complex subgroup, in which the latter condition may be missed.

Studies among PCPs demonstrated several barriers in identifying and managing early RA. These included limited understanding of typical symptoms, low confidence in detection, and reliance on imaging and blood test results prior to referral [41, 42]. A qualitative interview study among Danish PCPs reported an overarching theme of RA diagnosis as “like finding a needle in a haystack”, with increased difficulty in the absence of joint swelling, and varying trust in biomarkers [43]. A cross-sectional review identified that only 26% of British PCPs referred suspected RA without investigations, with an over-reliance on rheumatoid factor (RF) and inflammatory marker testing [44]. More than half of PCPs surveyed requested radiographs prior to referral, despite a low sensitivity for early RA [40]. Other studies have confirmed the reliance on blood tests including RF and showed that these influenced referral decisions, with negative results felt to exclude RA [31, 45].

Another aspect relevant to prompt specialist access is that of referral triage. Significant variation in the quality of Rheumatology clinic referrals has been identified in previous studies, and a reliable screening tool may help to better prioritise referrals for patients with IA [31, 35, 46]. This is particularly relevant in areas where Specialist services are limited, with applications in Telemedicine, and other remote-area services.

Our study has limitations, including the volume of data, which remains small for neural network development. The total number of cases analysed in the pilot and multicentre studies stands at 530, in addition to a background image library of 1013 labelled hand photographs. Ongoing data capture and algorithm development are planned, utilising one of the main advantages of ML in medical screening and diagnostics.

Other less frequent conditions, including gout and SLE, did not have sufficient case numbers for analysis. Gout does not commonly affect the hands and is often apparent from the presentation of podagra in the forefoot. If a library of tophaceous gout images can be incorporated into the photographic algorithm, this condition may be included in future application screening. Rarer causes of IA, such as SLE, would be expected to generate a screening result of IA, where the recommended specialist review would remain appropriate.

The clinical diagnosis by a Rheumatologist as the gold-standard outcome reflects current clinical practice. In the absence of more reliable objective measures, it remains the most appropriate comparison, as confirmed in other studies [47]. The expansion of analysis to include different Rheumatologists across Australia was designed to minimise single-user bias which could have affected the pilot study.

Following the completion of study recruitment, the App has been further developed to provide the point-of-care screening result to PCPs. Using a web-based server, the data inputs provide a result within 5 seconds of the competed process. For patients with isolated OA, methods for diagnostic confirmation with plain X-rays, and subsequent treatment recommendations, are provided. For those considered on screening to have IA, with or without background OA, a referral for specialist review is recommended. This could potentially be linked directly to a regional referral service.

The next stage of assessment for the App will entail a trial of real-world use in primary care settings. Participating PCPs will be able to use the App to obtain a real-time screening result for patients presenting with arthritis affecting the hands. Feedback will be sought on several elements of the user experience, including overall ease, speed, reliability, and recommendations for improvement.

## Conclusion

This multicentre validation study has confirmed the reliability of a smartphone application to identify different forms of arthritis affecting the hands. It is the first such tool to be able to identify coexistent IA and OA, through the combination of independent image processing and symptom questionnaire algorithms. It provides a rapid, easy to use, and reliable screening result for PCPs, and may help to improve the assessment, management and referral for patients presenting with various forms of arthritis. Such an approach could help to reduce the significant delays in referral and treatment for patients with IA, facilitate more accurate triage for specialist clinics, and improve PCP autonomy in confirming and managing isolated OA.

## Supplementary Information

**Additional file 1.**

## Data Availability

Our dataset is currently stored in a Google cloud account. The datasets generated and analysed during the current study are not publicly available due to potential infringement of the intellectual property regarding the algorithm development, but are available from the corresponding author on reasonable request.

## Abbreviations

ML: Machine learning
OA: Osteoarthritis
RA: Rheumatoid arthritis
PsA: Psoriatic arthritis
PCP: Primary care physician
CNN: Convolutional neural network
VGG: Visual geometry group
