# Supplementary material for: A multicentre validation study of a smartphone application to screen hand arthritis
Source: BMC Musculoskelet Disord. 2022 May 9;23:433. doi: 10.1186/s12891-022-05376-9 (PMC9081322; doi:10.1186/s12891-022-05376-9)
Supplement: Supplementary file 1 — Additional file 1. [file 12891_2022_5376_MOESM1_ESM.pdf]

Hollywood
Private Hospital
Part of Ramsay Health Care

Hollywood Private Hospital
ABN 36 003 184 889
Monash Avenue
Nedlands WA 6009
Telephone: 08 9346 6000
Facsimile: 08 9389 8470

hollywoodprivate.com.au

22 December 2021

Dr Mark Reed
Rheumatologist
Hollywood Rheumatology
56/85 Monash Avenue
Nedlands WA 6009

Sent via email: markreed@iinet.net.au

Dear Dr Reed

Re: Ethics review of study - ***A multicentre validation study to determine the validity of a smartphone application, for screening patients presenting with hand arthritis***

I write to confirm that as Chair of the Hollywood Private Hospital HREC in June 2019 I advised you that the above study presented no ethical issues and did not require formal ethics review by our HREC.

It was noted the purpose of this study was to validate a smart phone app for screening of arthritis of the hand using deidentified data. Patients consented to having a photograph taken of their hand. This minimal interaction involved no risk of harm or discomfort and negligible inconvenience to patients and no risk to their privacy

Kind regards

Terry Bayliss
Coordinator Development Projects and Research
Hollywood private Hospital
